# Reactive Spindle Cell Nodule in the Pancreas Post Fine Needle Aspiration: A Case Report and Literature Review

**DOI:** 10.7759/cureus.72666

**Published:** 2024-10-29

**Authors:** Milan R Akbari, Snehal Sonawane

**Affiliations:** 1 Pathology and Laboratory Medicine, UI Health/University of Illinois at Chicago, Chicago, USA

**Keywords:** atypical myofibroblastic tumors, inflammatory pseudotumors, postoperative spindle cell nodules, pseudosarcomatous myofibroblastic proliferation, reactive spindle cell nodules (rscns), spindle cell proliferation

## Abstract

Reactive spindle cell nodules (RSCNs) that occur after fine needle aspiration (FNA) are commonly documented in the literature. They are benign proliferation of spindle cells with some mitotic figures and nuclear pleomorphism that arise after tissue injuries like FNA. These lesions are non-capsulated and surrounded by parenchyma of organ tissue. Although RSCNs after FNA can occur in various organs of the body, there is a lack of well-established studies describing their presence in the pancreas. We present a case of a 74-year-old female treated with pancreatectomy for intraductal papillary mucinous neoplasm (IPMN), and RSCNs were found incidentally during post-surgery microscopic evaluation. This lesion showed similar characteristic microscopic features of the RSCNs found at different anatomical sites. To the best of our knowledge, this may be the first case of RSCNs after FNA into the pancreatic. This case expands the diagnostic framework of pancreatic spindle cell tumors.

## Introduction

Reactive spindle cell nodules (RSCNs) are rare, benign proliferative lesions of spindle cells with some mitotic figures and nuclear pleomorphism that often arise in response to tissue injuries [1,2]. RSCNs are also known as atypical myofibroblastic tumors, postoperative spindle cell nodules, inflammatory pseudotumors, and pseudosarcomatous myofibroblastic proliferation [1,3-5]. While these nodules can occur at different anatomical locations, including in the abdomen, thyroid, genitourinary system, lymph node, prostate, head and neck, and breast, their occurrence in the pancreas is not reported [1,2,4,6-8]. These nodules are composed of spindle cells and commonly stained positive to immunohistochemical stains vimentin, smooth muscle actin (SMA), and desmin, suggesting the myofibroblastic origin of spindle cells [1,3,9,10].

FNA is an extensively used diagnostic procedure for evaluating various masses, including pancreatic mass [1,2,6,7,11-13]. A wide range of post-FNA changes from hemorrhage to RSCNs in the tissue parenchyma are well described in the English literature [1,2,13,14]. Due to the nuclear pleomorphism and mitotic figures, RSCNs mimic sarcomatous tumors and can easily mislead erroneous histopathological examination and diagnosis [1,2,7,10,12,15,16]. We have encountered a similar lesion in the pancreas of a 74-year-old female who had previously undergone an FNA procedure. The microscopic findings of the lesion are consistent with the features of RSCNs described in previously published articles.

## Case presentation

**Figure 1:**
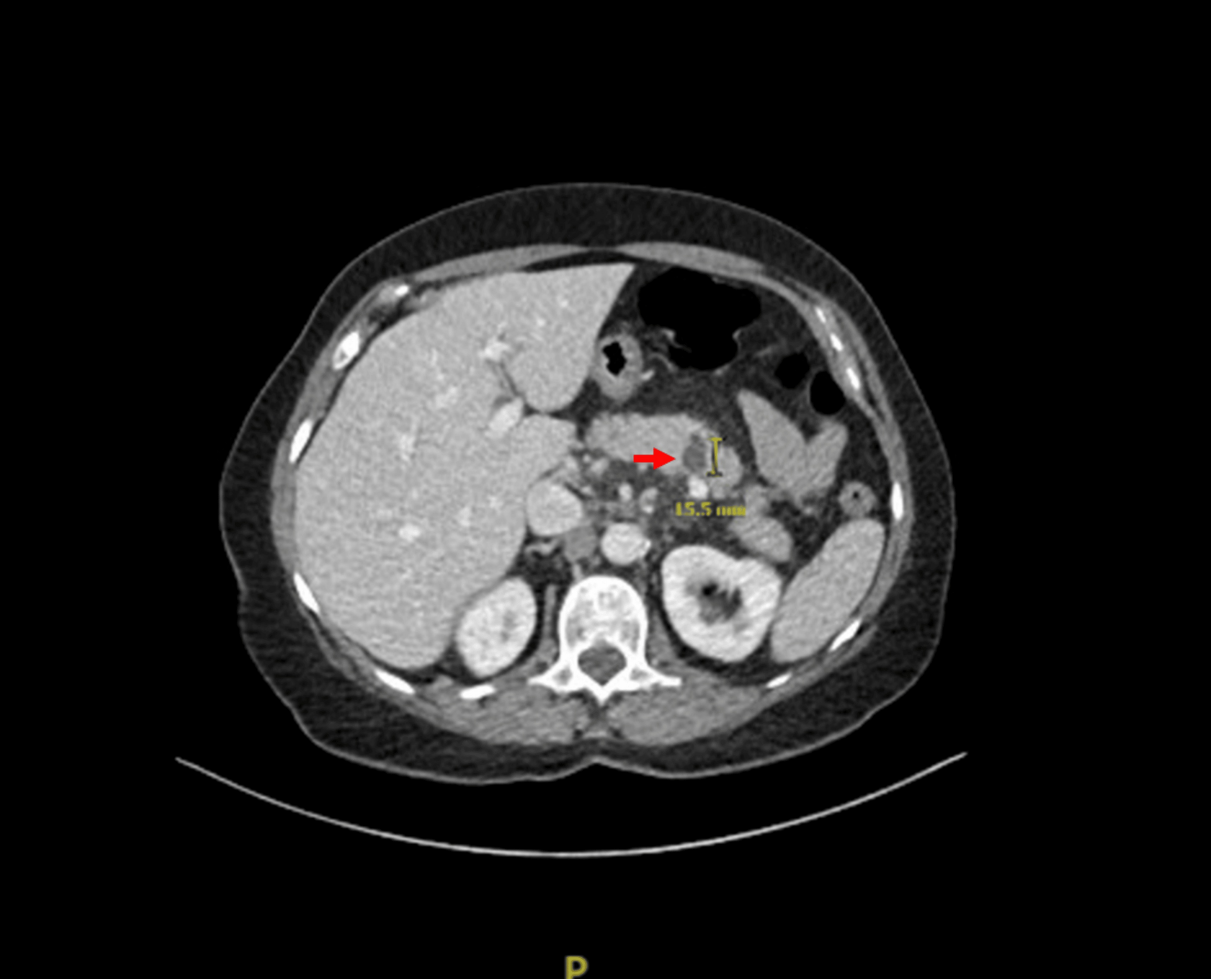
CT scan of abdomen showing 1.6 cm cystic lesion (red arrow) in the mid-pancreatic body.

A 74-year-old female presenting with a previous medical history of gastroesophageal reflux disease, coronary artery disease, and hypertension was evaluated for a cystic pancreatic mass found on CT. The lesion was a cystic 1.6 cm mid-pancreatic body lesion likely representing an intraductal papillary mucinous neoplasm (IPMN) (Figure 1). She had an episode of pancreatitis and was hospitalized for several days two months ago. There were neither personal risk factors nor personal history of IPMN.

**Figure 2:**
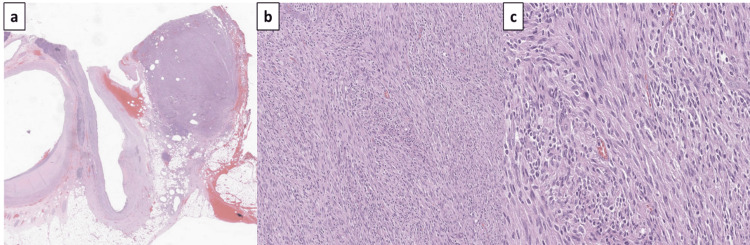
H&E stain: (a) well-circumscribed unencapsulated lesion (b) spindle cell lesion (magnification, x200) (c) spindle cell lesion with some nuclear pleomorphism and very few mitotic activities, multinucleated giant cells, and thinned blood vessels (magnification, x400). H&E: hematoxylin and eosin

She underwent endoscopic ultrasound (EUS) with FNA during hospitalization. Based on the pathological finding, the cystic mass was considered an IPMN with a positive KRAS mutation suggestive of high risk for malignancy, and she was planned for partial pancreatectomy after two months. Microscopic examination during post-surgery histopathological evaluation revealed a well-circumscribed, unencapsulated lesion primarily composed of spindle cells with mild nuclear pleomorphism, minimal mitotic activity, occasional inflammatory cells, and thin-walled blood vessels (Figure 2).

**Figure 3:**
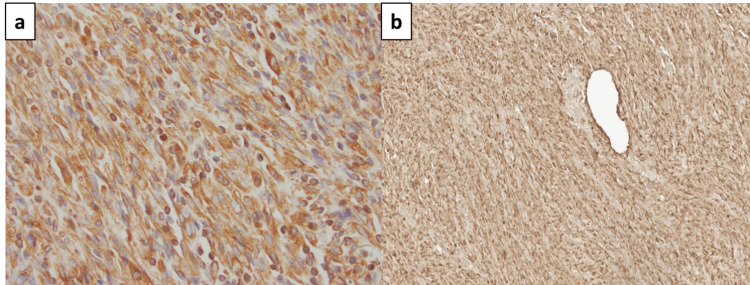
IHC stains: (a) vimentin positive the spindle cells (magnification, x400); (b) CD68 positive IHC staining (magnification, x200). IHC: immunohistochemistry

Immunohistochemistry (IHC) studies were performed with appropriate controls. Immunostains for the expression of vimentin and CD68 were positive (Figure 3), while DOG-1, SMA, desmin, AKL-1, CAM 5.2, inhibin, CD10, CD117, CD138, and CD10 were negative. The Ki-67 index was 3%, consistent with the benign nature of the lesion.

## Discussion

FNA is a widely used diagnostic procedure for the evaluation of various masses [1,2,6,7,11,13]. Post-FNA changes have been classified into two groups: acute and chronic [2,13,14]. Acute changes occur within three weeks of tissue insults and involve hemorrhage, inflammatory cell infiltration, giant cells, and necrosis [13,14]. Chronic changes take place after three weeks of tissue injuries and include inflammatory changes, infarction, necrosis, fibrosis, spindle cell proliferation, nodular formation, capsular distortion (pseudo-capsular invasion), and replacement tissue parenchyma of organs or tumors [1,13,14]. RSCNs are rare, benign proliferative lesions of spindle cells with some mitotic figures and nuclear pleomorphism that often arise in response to tissue injuries like FNA, surgery, and trauma [1,8,11,13,14]. These changes have been well described in the different parts of the body in published articles. However, to our knowledge, this may be the first case of RSCNs in the pancreas. The pathophysiology of RSCNs is not well established yet, but it may be considered reactive hyperproliferation of fibroblast or myofibroblast in response to the tissue injury.

RSCNs are relatively circumscribed and unencapsulated small nodules and are mainly composed of spindle-shaped cells with elongated nuclei and ill-defined cytoplasmic borders [1,3,10,11,17]. In addition to spindle cells, RSCNs also contain small thinned wall blood vessels, fibrous stroma, and some inflammatory cells [3,6-8,10,11,15,17]. RSCNs can mimic malignant spindle cell sarcomatous tumors due to their histological appearance of spindle cells with brisk mitotic figures and some nuclear pleomorphism [1-3,7-9,11]. This appearance poses a diagnostic challenge to pathologists.

IHC plays an important role in identifying such lesions. RSCNs commonly show positive staining for IHC markers smooth muscle actin (SMA), vimentin, and desmin, indicating a myofibroblastic origin of the spindle cells. In contrast, equivocal results are observed with AE1/AE3 and Cam5.2 staining, while ALK-1 and EMA stain negative [1,3,4,8,11,12,18,19]. Positivity to ALK stain and ALK gene rearrangement was reported in approximately 50% of cases, suggestive of malignant RSCNs [3,12]. IHC stain Ki67 helps to differentiate it from malignant tumors, as RSCNs show a lower mitotic count than malignant tumors. The results of various IHC stains were documented in previously published literature (Table 1). The table shows that RSCNs are not always positive for SMA and desmin but are almost always positive for vimentin. The lesion in this case was also found negative to immunostains SMA and desmin while positive to vimentin.

**Table 1:** Immunohistochemical reactivity of RSCNs in various studies. ND: no data, VA: variable, RSCN: reactive spindle cell nodules.

Study	Vimentin	SMA	Desmin	ALK-1	CAM5.2	CD68
Baloch et al., 1999 [1]	ND	10/10	ND	ND	ND	VA
Gobbi et al., 2000 [2]	7/7	7/7	ND	ND	ND	0/5
Montgomery et al. 2006 [3]	ND	23/35	15/19	20/35	10/15	ND
Coffin et al., 1995 [4]	74/75	68/74	51/74	ND	27/74	ND
Wick et al., 1998 [5]	ND	ND	2/2	ND	2/2	ND
Lo et al., 1992 [6]	1/1	0/1	0/1	ND	ND	ND
Huang et al., 1990 [7]	3/3	ND	2/3	ND	ND	ND
Kim et al., 2013 [11]	1/1	1/1	1/1	0/1	ND	ND
Kang et al., 2013 [12]	1/1	1/1	0/1	0/1	0/1	ND
Zhao et al., 2014 [15]	1/1	1/1	ND	ND	ND	ND
Iczkowski et al., 2001 [16]	4/4	2/4	2/3	ND	ND	ND
Garijo et al., 2008 [18]	1/1	1/1	0/1	0/1	0/1	ND
Harik et al., 2006 [19]	ND	23/34	21/35	12/26	ND	ND
This study	1/1	0/1	0/1	0/1	0/1	1/1

The histological features of the lesion in this case are similar to the RSCNs described in various organs of the body. History of an FNA biopsy and this lesion's small size, proximity to the FNA biopsy site, low mitotic count, presence of inflammatory cells, and negative immunostaining of malignant tumor helped differentiate it from sarcoma or sarcomatoid malignancy.

Differential diagnoses of RSCN following FNA in the pancreas include leiomyoma, gastrointestinal stromal tumor (GIST), leiomyosarcoma, metastatic sarcoma, schwannoma, malignant fibrous histiocytoma, granular cell tumor, and neuroendocrine carcinoma [20]. IHC stains help to differentiate it from other conditions, as RSCNs typically show negative results to IHC stains used to evaluate malignant tumors. It is important to identify these post-procedural benign changes to avoid aggressive treatment and misdiagnosis of malignant conditions of the pancreas.

## Conclusions

We have described a patient with reactive spindle cell nodules that occur in the pancreas after the FNA, which was performed for further evaluation of imaging results and was indicative of another lesion of the pancreas. Despite the rarity of post-FNA RSCN in the pancreas, it should be considered as a differential diagnosis of pancreatic masses at the procedural sites. We conclude that pathologists should be aware of these post-FNA cytological changes in the pancreas. The following features may help to differentiate RSCNs from sarcoma or sarcomatoid malignancy: relatively small size, history of tissue insult, low mitotic activity, presence of needle tract in FNA-related RSCN, inflammatory cell infiltrate, and IHC stains.
